# Regional Anesthesia for Postoperative Pain Control

**DOI:** 10.1155/2014/309606

**Published:** 2014-06-18

**Authors:** Ahmet Eroglu, Engin Erturk, Alparslan Apan, Urs Eichenberger, Ozgun Cuvas Apan

**Affiliations:** ^1^Department of Anesthesiology and Intensive Care Medicine, Karadeniz Technical University School of Medicine, 61000 Trabzon, Turkey; ^2^Department of Anesthesiology and Intensive Care Medicine, Giresun University School of Medicine, Nizamiye Yerleskesi, Orhan Yilmaz Cad. Mumcular Sok. No. 1, 28200 Giresun, Turkey; ^3^Department of Anesthesiology and Pain, Bern University Hospital, 3000 Bern, Switzerland

Pain is an outstanding problem after surgical trauma. Pain following surgery may initiate variety of mechanisms including inflammatory, visceral, or somatic in origin and may persist to be chronic pain if improperly treated. The incidence of postoperative pain has been reported to be as higher as 60%, and, despite intensive effort, it is not able to resolve completely [1].

Most of the surgeries become less invasive and are increasingly being outpatient-based in time dependent manner. Besides technical developments on surgery, this tendency is mainly dependent on effective pain control and reduction of the side effects related to the treatments. Opioids and nonsteroidal anti-inflammatory drugs are the other main components of pain therapy which have well-known side effects that may limit their use. The modern concept of pain treatment includes multimodal approach and mainly targets to decrease opioid use in combination with other drugs or techniques in order to reduce drug related side effect profile especially to prevent postoperative respiratory depression.

Until recently, there is no convincing data to demonstrate the beneficial effects of regional anesthesia on postoperative mortality, cardiovascular complications, or the incidence of thromboembolism when thromboprophylaxis is concomitantly in use, but all authors indicate that it may decrease the morbidity including postoperative pulmonary complications after major abdominal surgery and may improve the patient recovery after orthopedic surgery. It is also demonstrated that regional anesthesia reduces the incidence of postoperative pain, opioid consumption, and related side effects such as nausea and vomiting [2].

Central neuraxial blocks alone or in combination with catheter techniques are performed in various surgical interventions in order to decrease surgically induced stress and inflammation, improve pulmonary functions, and reduce the period for ambulation with better pain control. In a meta-analysis, it has been stated that postoperative pain control with local anesthetic infusion with long term catheter placement demonstrated a decrease in the occurrence of chronic pain [3].

Peripheral nerve blocks are the other type of regional techniques. Improvement in ultrasound technology may increase clinical applications for peripheral nerve and truncal blocks. Real time ultrasound use while performing the block may reduce the complications, performance time, and local anesthetic requirements. It also provides reappraising the older techniques with carrying potential complications. The rate of success may increase with clinical experience. Peripheral nerve blocks seem to lack systemic side effect related to sympathetic blockade and lesser incidence of minor complications including urinary retention when compared with central neuraxial blocks or catheter applications. Peripheral nerve blocks seem to be safer than either central neuraxial blocks or general anesthesia, especially in patients with severe coexisting disease [2].

In this special issue, we focused on the clinical studies and review articles related to various aspects of regional anesthesia for postoperative pain control. Some of these studies have investigated the effects of additives combined with local anesthetic mixture on postoperative analgesia in regional intravenous anesthesia. One of these reports investigated two additives, namely, ketamine or tramadol, combined with ropivacaine. While onset and duration of motor and sensorial block were shorter, the period of analgesia was longer in the tramadol group in the paper entitled “*Does the addition of tramadol and ketamine to ropivacaine prolong the axillary brachial plexus block?*” On the same topic, the efficacy on nitroglycerine or lornoxicam combination with lidocaine for regional intravenous anesthesia was searched. Each of these drug combinations effectively increased the tolerance to the tourniquet and decreased pain during peri- and postoperative period, which is discussed in the paper entitled “*Peri- and postanalgesic properties of lidokain, lornoxicam, and nitroglycerine combination at intravenous regional anesthesia*.” Likewise, dexketoprofen or paracetamol combination with lidocaine was compared to each other for the same purpose. Addition of dexketoprofen increased the duration of motor block and decreased pain scores, and lesser analgesic consumptions were observed in groups with paracetamol or dexketoprofen when compared with the control, in the paper entitled “*Comparison of the effect of lidocaine adding dexketoprofen and paracetamol in intravenous regional anesthesia*.”

Central neuraxial blocks including spinal and epidural anesthesia were the subjects of the other studies. The effects of intraoperative intravenous magnesium sulfate infusion on sensorial and motor block characteristics and postoperative pain scales in female patients undergoing abdominal hysterectomy under spinal anesthesia were investigated. Authors indicated that the sensorial block period of spinal anesthesia increased, and better pain scores were observed with magnesium therapy without significant complications, in the paper entitled “*The effect of intravenous magnesium sulfate infusion on sensory spinal block and postoperative pain score in abdominal hysterectomy.*” In the other report, the difference of spinal block characteristics with levobupivacaine 0.5% plain solution at room and body temperature (23°C and 37°C) was observed in male patients undergoing transurethral resection of prostate operation. Authors indicated that the use of 0.5% levobupivacaine spinal anesthesia heated to the body temperature accelerated the start of sensory and motor block in the paper entitled “*The effects on sensorial block, motor block, and haemodynamics of levobupivacaine at different temperatures applied in the subarachnoid space.*”

Epidural anesthesia was the main topic for postoperative pain control in two clinical trials. The influence of preemptive local anesthetic infusion with a thoracic epidural catheter on thoracotomy was evaluated and the effects of preemptive and postoperative infusions were compared. It was shown that preemptive administration of local anesthetic solution offered superior analgesic quality and lesser analgesic consumption, which is shown in the paper entitled “*The effectiveness of preemptive thoracic epidural analgesia in thoracic surgery*.” In a retrospective study, the difference of analgesic efficacy of epidural anesthesia was compared with total intravenous anesthesia performed with propofol and remifentanil infusion in patients who underwent abdominal aortic aneurysm repair. It was stated that the quality of analgesia improved with epidural anesthesia, and enteral nutrition was performed earlier, in the paper entitled “*Efficacy of continuous epidural analgesia versus total intravenous analgesia on postoperative pain control in endovascular abdominal aortic aneurysm repair: a retrospective case-control study.*”

Caudal anesthesia is commonly performed in pediatric patients for surgical anesthesia and postoperative analgesia. In a clinical report entitled “*The effects of single-dose rectal midazolam application on postoperative recovery, sedation, and analgesia in children given caudal anesthesia plus bupivacaine*,” the effects of rectal midazolam combined with caudal anesthesia on the quality of sedation and postoperative analgesia were investigated, but no significant contribution was demonstrated.

Gabapentin, a drug that is used for treatment of neuropathic pain, has also been investigated for possible effects on postoperative analgesia. In a review entitled “*Gabapentin in acute postoperative pain management,*” the influence of gabapentin treatment on postoperative pain control was documented and it was found that gabapentin was an efficacious agent for postoperative analgesia in various types of surgery.

Pain is a common problem in all age groups of patients. Postoperative analgesia is a developing area and regional anesthesia is an essential part of this treatment. It is worthy of noting that future studies and technical developments about regional anesthesia will contribute vital advancements to postoperative pain control.

*Ahmet Eroglu* *Engin Erturk* *Alparslan Apan* *Urs Eichenberger* *Ozgun Cuvas Apan*
